# In-Air Evoked Potential Audiometry of Grey Seals (*Halichoerus grypus*) from the North and Baltic Seas

**DOI:** 10.1371/journal.pone.0090824

**Published:** 2014-03-14

**Authors:** Andreas Ruser, Michael Dähne, Janne Sundermeyer, Klaus Lucke, Dorian S. Houser, James J. Finneran, Jörg Driver, Iwona Pawliczka, Tanja Rosenberger, Ursula Siebert

**Affiliations:** 1 Institute for Terrestrial and Aquatic Wildlife Research, University of Veterinary Medicine Hannover, Foundation, Büsum, Schleswig-Holstein, Germany; 2 Institute for Marine Resources and Ecosystem Studies, Imares, Wageningen UR, Den Burg, North Holland, The Netherlands; 3 National Marine Mammal Foundation, San Diego, California, United States of America; 4 U.S. Navy Marine Mammal Program, Space and Naval Warfare Systems Center Pacific, San Diego, California, United States of America; 5 Veterinary Clinic, Reinsbüttel, Schleswig-Holstein, Germany; 6 Hel Marine Station, University Gdansk, Hel, Pomerania, Poland; 7 Seal Centre Friedrichskoog, Friedrichskoog, Schleswig-Holstein, Germany; University of South Florida, United States of America

## Abstract

In-air anthropogenic sound has the potential to affect grey seal (*Halichoerus grypus*) behaviour and interfere with acoustic communication. In this study, a new method was used to deliver acoustic signals to grey seals as part of an in-air hearing assessment. Using in-ear headphones with adapted ear inserts allowed for the measurement of auditory brainstem responses (ABR) on sedated grey seals exposed to 5-cycle (2-1-2) tone pips. Thresholds were measured at 10 frequencies between 1–20 kHz. Measurements were made using subcutaneous electrodes on wild seals from the Baltic and North Seas. Thresholds were determined by both visual and statistical approaches (single point F-test) and good agreement was obtained between the results using both methods. The mean auditory thresholds were ≤40 dB re 20 µPa peak equivalent sound pressure level (peSPL) between 4–20 kHz and showed similar patterns to in-air behavioural hearing tests of other phocid seals between 3 and 20 kHz. Below 3 kHz, a steep reduction in hearing sensitivity was observed, which differed from the rate of decline in sensitivity obtained in behavioural studies on other phocids. Differences in the rate of decline may reflect influence of the ear inserts on the ability to reliably transmit lower frequencies or interference from the structure of the distal end of the ear canal.

## Introduction

Grey seals (*Halichoerus grypus*) are a phocid seal species with an increasing population in the North Sea on Heligoland and in the Wadden Sea of Lower Saxony and Schleswig-Holstein [1], [2], [3]. In the Baltic Sea, increasing observations of grey seals have been recorded along the coast of Mecklenburg-Western Pomerania since the 1980’s [4]. However, the status of the grey seal according to the latest national report for the habitats directive (reporting period 2000–2006) was ‘unfavourable/inadequate’ for the Atlantic biogeographic region (German North Sea) and ‘unfavourable/poor’ for the continental biogeographic region (German Baltic Sea; [5], for full EU report see [6]).

Grey seals inhabit areas subjected to substantial anthropogenic influence [7]. Due to their amphibious nature, they are confronted with human activities both above and below the water surface. Interactions with human activity are anticipated to increase as plans to build large numbers of offshore wind turbines (OWTs) in the North and Baltic Seas have been established in Germany and neighbouring countries [8]. In Germany, 25 GW of capacity are planned to be installed by 2030 [9] resulting in the construction of 5,000 turbines, or approximately 63 wind farms of 80 turbines each. As a result, concern about the effect of acoustic emissions on grey seals during construction and operation of OWTs has increased, as it has for many species of marine mammals [10], [11], [12], [13].

Grey seals use acoustic signals for communication purposes both in air and under the water. In air, they rarely produce communication signals [14], but underwater they exhibit complex vocal repertoires [15]. Seals may not be vitally dependent on their auditory system to survive as adults [16], but sound production and reception are critical for mother-pup affiliation and for mating displays [17], [18]. Seals can vocalize across a wide frequency range, although with few exceptions most calls are low frequency in nature [19], [20]. Sounds produced by grey seals are typically less than 3 kHz, although click type sounds have been recorded with harmonic content as high as 15–30 kHz [15], [21], [22].

The construction of OWTs or their operation can possibly induce stress, mask communication signals, or temporarily or permanently impact hearing in grey seals. The effects vary depending on the source level and frequency content of the signal, its duty cycle, the distance of the source to the animal, and the animal’s frequency range of hearing and hearing sensitivity [23], [24], [25]. Consequently, important considerations for the construction and operation of wind farms near grey seal habitats are the source level and frequency content of construction and operational sounds, the hearing range and sensitivity of grey seals, and the potential impact of these sounds on the seals.

Nearly 40 years ago, a single investigation on the hearing ability of grey seals was conducted with in-air and underwater sounds [26]. The study indicated that the most sensitive frequency for the grey seal in air was around 4 kHz, which is within the range of reported best sensitivities for other phocid seals in air [27], [23]. Three data points from a former study [28] suggested that grey seals have better hearing abilities in air than reported by Ridgway and Joyce [26]. However, continued tests on the grey seals were not successful as the grey seals closed their ear canals in the presence of acoustic test signals by manipulating an external auditory sphincter, i.e. regular headphones or loudspeakers were unsuccessfully used.

The study described here aimed to gain further knowledge on the hearing sensitivity of grey seals by employing earphone inserts. The procedure prevents the seals from manipulating the external auditory sphincter and therefore interfering with sound reception. The study demonstrates that ear inserts can be used to study the hearing of sedated seals and may be a feasible approach to addressing issues of manipulation of the external ear canal by phocid seals.

## Methods

### Ethics Statement

Auditory threshold measurements on grey seals at the Seal Centre Friedrichskoog (Germany) were conducted under a permit held by the German Ministry of Energy, Agriculture, the Environment and Rural Areas Schleswig-Holstein (permit number: 42-4/09; address: Abteilung V 2, Referat 24 - Tierschutz - Mercatorstraße 5, 24106 Kiel). Studies conducted at Hel Marine Station (Poland) were performed under permission from the General Director for Environmental Protection (GDOŚ/DOPPozgiz-4200-23/2139/10/ls; address: ul. Wawelska 52/54, 00-922 Warszawa ). All experiments were performed in accordance with institutional and national laws and ethical principles.

### Subjects

Grey seals were examined at the Seal Centre Friedrichskoog e.V., Germany (two individuals), and Hel Marine Station, University of Gdansk, Poland (four individuals). Five of the animals were males and one was a female. The seals ranged from twelve to sixteen weeks of age and ranged from 33.3–59.0 kg. Grey seals tested at the Seal Centre Friedrichskoog e.V. were initially found along the German coast of Schleswig-Holstein and Heligoland and taken into rehabilitation either because of abandonment/loss of mother or because of their poor health status. Animals held at the Hel Marine Station were born at the station and released after the weaning period. These grey seals were sedated to fit telemetry devices before release and the sedation was extended for auditory brainstem response (ABR) measurements.

### Sedation

The health of each seal was evaluated by an experienced veterinarian prior to measuring auditory thresholds and audiometry was performed only on those animals deemed to be in good health. Animals were sedated with an intramuscular injection of midazolam (0.2 mg/kg; 15 mg/3 ml, HEXAL, Germany) and ketamine (1.5 mg/kg; 100 mg/ml, Albrecht, Germany) or tiletamine/zolazepam (1.5–2 mg/kg; 100 mg/ml, VIRBAC, Germany). Sedation was augmented when the animals showed irregular short latency evoked potentials or signs of awareness. The time given to the hearing measurements did not exceed 90 minutes in duration per individual.

### Stimulus Presentation and ABR Measurements

**Figure 1:**
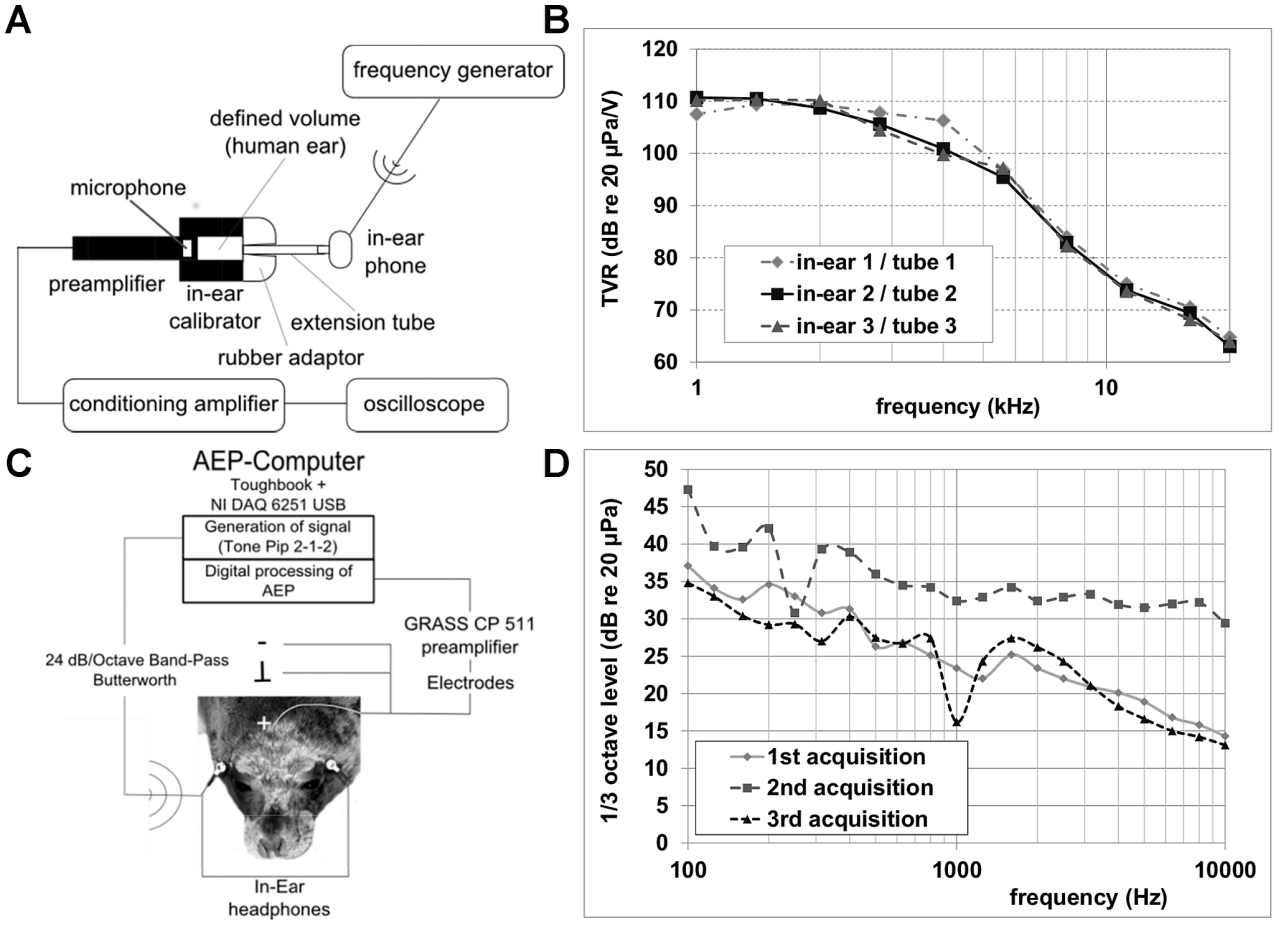
Setups, configuration and calibrations. **A.** Calibration setup for headphones and ear insert combinations; **B.** Results of calibrations in terms of transmit voltage response (TVR) over the tested frequency range; **C.** Configuration of the measurement system for ABRs collected from grey seals wearing the headphones and inserts. For more details on electrode placement, see Houser et al. [41] for an example on northern elephant seals (*Mirounga angustirostris*); **D.** Third-octave levels of background noise during the different data acquisitions (1^st^ acquisition: FR01; 2^nd^ acquisition: FR02; 3^rd^ acquisition: all HL animals).

Stimulus presentation and ABR measurements were conducted with the Evoked Response Study Tool (EVREST; [29], [30]). Sounds were presented with an earphone insert to prevent the animal from closing the ear canal. The earphones (type SE-72, Monacor, Germany), with the inserts used to hold the ear canal opened in place, were calibrated using a frequency generator (type 33220A, Agilent, USA), an artificial ear (type 4157, Brüel & Kjær, Denmark) connected to a calibrated microphone (type 2669, Brüel & Kjær), and a conditioning amplifier (type NEXUS 2690, Brüel & Kjær), shown in Figure 1A. The results of calibration in terms of transmit voltage response (TVR) over the tested frequency range is shown in Figure 1B. The outer diameter of the custom-made tubular ear insert was 5 mm and the inner diameter was 3.8 mm. The length of the ear insert was standardized to 22 mm to prevent any damage to the ear drum and was chosen based on measurements of the outer ear canal taken during necropsies of deceased grey seals.

Test signals consisted of 5-cycle tone pips (2-1-2) ranging from 1 kHz to 20 kHz in octave or half-octave steps (10 frequencies in total). Signals were generated using a Toughbook computer (Panasonic) in combination with a USB data acquisition board (NI USB 6251, National Instruments, USA). Signals were band-pass filtered (100 Hz –250 kHz, 24 dB/octave; Krohn-Hite, USA) before transmission to the earphone (Figure 1C). Tone pips were generated with a 1 MHz sampling rate at 16-bit resolution and presented at a rate of 58.8/s with a linear rise and fall over the first and last two cycles of the tone pip, respectively. Signals were attenuated via custom-designed attenuators. Each stimulus was calibrated by calculating its peak equivalent sound pressure level (peSPL).

Brainstem responses were recorded using sub-dermal needle electrodes (NE-224S, Nihon Kohden, Japan) placed at three positions along the dorsal midline of the grey seals (Figure 1C). Based on previous comparative measurements that indicated the placement for obtaining the best response, the active (+) electrode was placed at the vertex of the head, the inverting electrode (−) between the scapulae, and the ground electrode was inserted at the neck [31]. The ABRs were amplified and filtered (0.3–3 kHz) with a bio-potential amplifier (IP511, Grass Technologies, USA), digitized at 50 kHz via the NI USB 6251, and synchronously averaged. For each stimulus presentation, a total of 1024 epochs were collected. Background noise was measured using a sound level meter (type XL 2, NTi audio, Switzerland) at the time of the ABR measurements.

### Data Analysis

**Figure 2:**
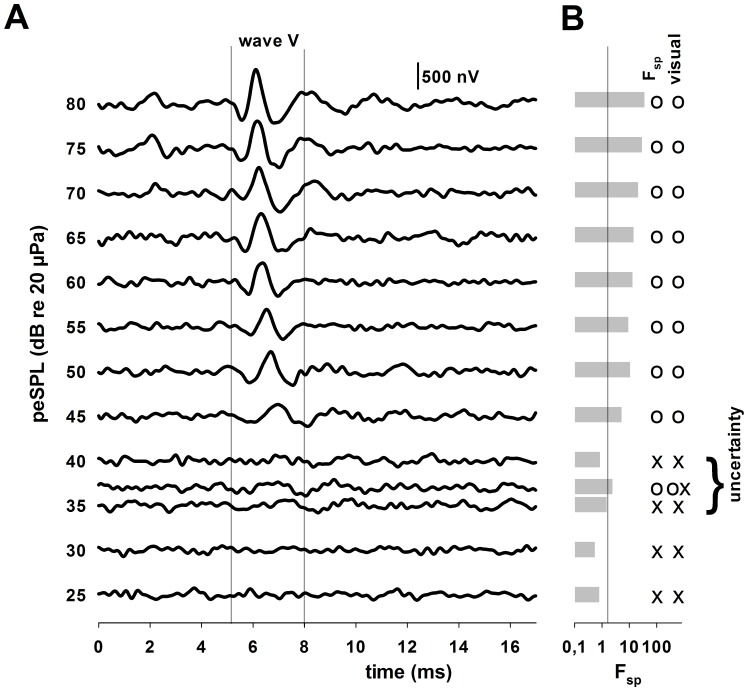
Evoked potentials and analysis. **A.** ABR measurement data of subject FR02. The evoked potentials at 16_sp_-test; **B.** Results of the F_sp_-test (bar plot, estimated variance of the ABR and the background noise as critical value for the test) and visual determination of the hearing threshold are indicated to the right of the panel. The waveform is classified as to whether the ABR is detected (o) or undetected (x) for F_sp_ and visual verification, respectively.

The ABR waveforms were characterized according to the nomenclature of Jewett and Williston [32]. Attenuation in the amplitude of wave V of the evoked response was used to determine the threshold for a given stimulus frequency (Figure 2A). Waveforms were visually evaluated according to decreasing stimulus amplitude. A detected ABR was defined as a waveform showing a clearly visible wave V, while those without an obvious wave V were considered as undetected. Thresholds were defined as the midpoint between the stimulus level corresponding to the last waveform detected and the highest stimulus level where no waveform was detected (Figure 2A).

An alternative, statistical approach to determining the threshold was conducted for comparison purposes. This approach is the single point F-test (F_sp_) described by Finneran [29] and by Elberling and Don [33] for the quality estimation of averaged auditory brainstem responses. The method calculates the ratio between the estimated variance of the auditory brainstem response (ABR) and the estimated variance of the background noise (results in Figure 2B). The degrees of freedom (*v_1_, v_2_*) required for determining the critical value of the statistical test correspond to the degrees of freedom in the numerator and denominator of the statistic. Whereas the degrees of freedom in the denominator are equal to the total number of accepted sweeps, it is difficult to evaluate the degrees of freedom for the numerator from the actual test recordings [33]. Therefore, for the tests conducted here, it was assumed that the degrees of freedom in the numerator were equal to the samples in the analysis window. Using this approach, the resulting threshold for a stimulus frequency was defined as the midpoint between the lowest stimulus level at which the ABR was detected and the highest stimulus level at which it was not detected.

## Results

### Background Noise

Hearing tests were performed on three occasions and noise levels, calculated across 1/3 octave bands, were recorded at the time of the tests. Background noise levels during the hearing tests were generally low (Figure 1D). However, during the second set of hearing tests (subject FR02) levels were elevated by ∼10 dB across the range of frequencies measured.

### Impact of the in-ear Headphones with Inserts

**Figure 3:**
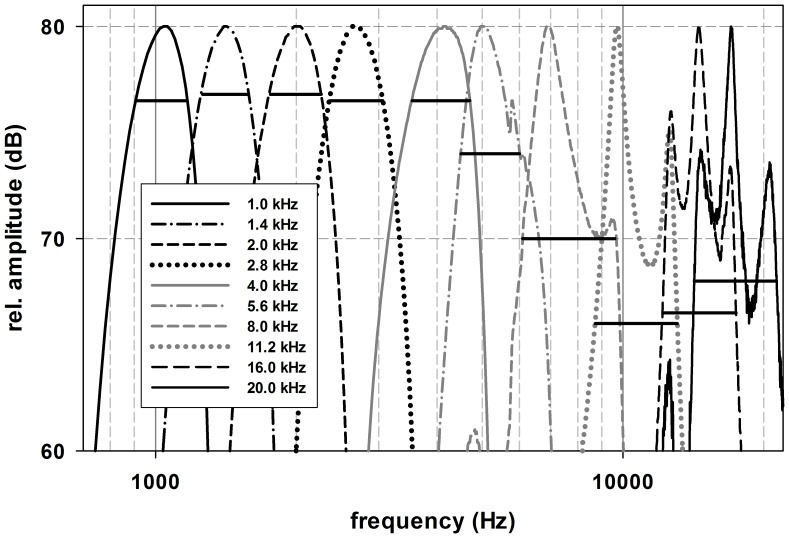
Spectra of the stimulus tone pips. Spectra of the generated (2-1-2) tone pip stimuli for the desired centre frequencies measured with the artificial ear. The peak frequencies showed a shift to frequencies lower than the desired peak frequencies for tone pips with centre frequencies ≥5.6 kHz. Signal distortion worsened and the bandwidth became more asymmetric relative to the centre frequency as the centre frequency of the tone pip increased.

**Figure 4:**
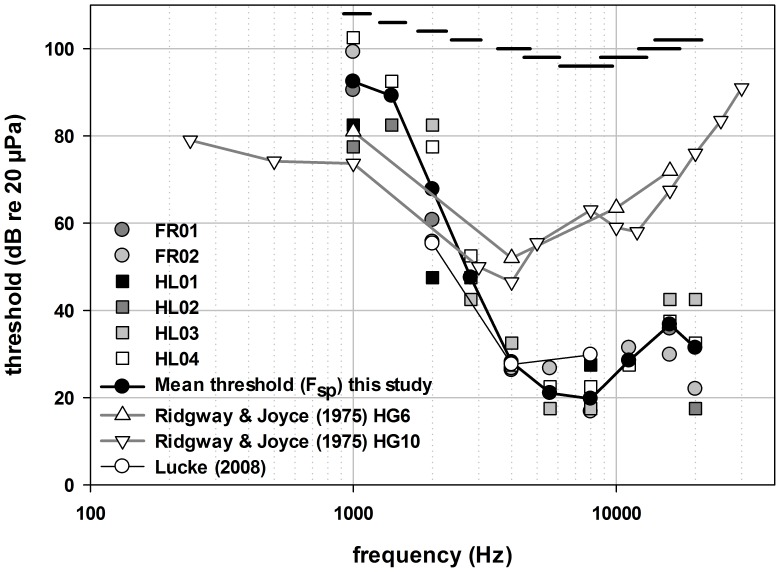
Results for in-air ABR thresholds on grey seals. In-air ABR thresholds derived by an F_sp_-test for six grey seals exposed to acoustic stimuli via earphone inserts (symbols without lines) and the mean of these results (black line, black filled circles). The horizontal black lines indicate the bandwidth relative to the centre frequency of each test stimulus. The results are provided in comparison to results from a study using cortical evoked potentials in air ([26]; grey filled triangles, grey lines) and a study using ABRs with calibrated headphones on a wild grey seal ([28]; open circles, black line).

**Figure 5:**
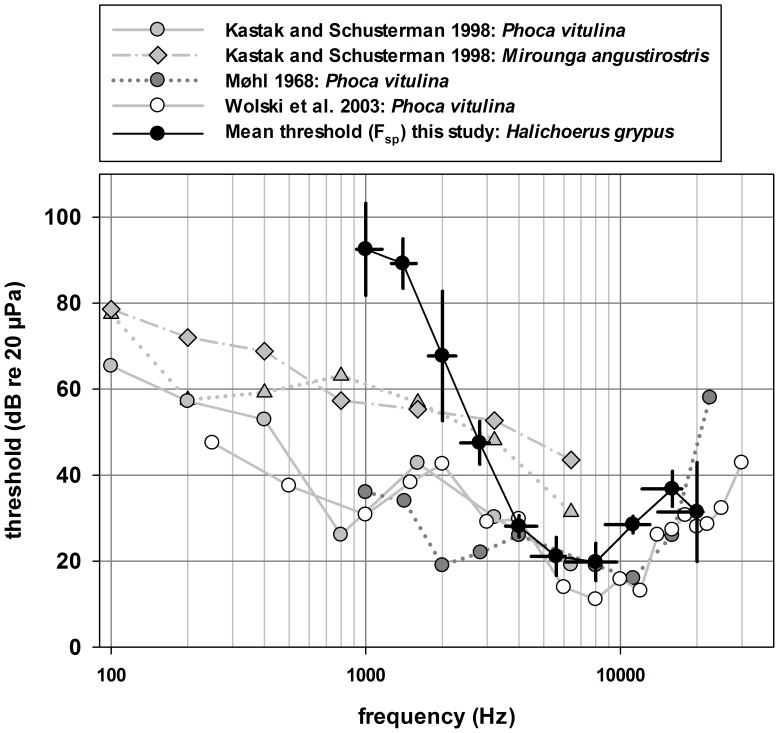
Behavioural thresholds of different seal species and the ABR thresholds of this study. In-air behavioural hearing thresholds from the northern elephant seal (*Mirounga angustirostris*; [36]) and harbour seal (*Phoca vitulina*; [36], [42], [27]) compared to the in-air thresholds obtained from ABR measurements on grey seals (*Halichoerus grypus*) in this study. The horizontal black lines indicate the bandwidth relative to the centre frequency of each test stimulus.

Spectral analysis of signals transmitted to the ear of the seal via the inserts demonstrated a distortion in the signal that became progressively worse with increasing centre frequency (Figure 3). Specifically, for tone pips with centre frequencies ≥5.6 kHz, the peak frequency within the spectra began to shift to frequencies lower than the desired centre frequency. As the distortion of the signal increased, the bandwidth of the signal also increased. Following the procedures of Wolski et al. [27], we calculated the −3 dB stimulus bandwidth relative to the amplitude at the centre frequency of the signal (Figure 3). With increasing frequency, the bandwidth relative to the centre frequency became more asymmetric (horizontal black lines in Figure 4 and horizontal error bars in Figure 5).

### ABR Measurements

Figure 2A shows a typical attenuation series of ABR waveforms. The responses depicted in Figure 2A are from a tone pip with a centre frequency of 16 kHz and a maximum peSPL of 80 dB re 20 µPa. Wave V is clearly visible for amplitudes above 45 dB and the amplitude of wave V decreases into the background noise with decreasing sound pressure level. Hearing thresholds determined from the ABR attenuation series using the F_sp_ are shown for the grey seals in Figure 4. Seals were most sensitive within the frequency range of 4–11.2 kHz where thresholds were below 40 dB peSPL. For 5.6 and 8 kHz, thresholds approached or fell below 20 dB peSPL. For most of the frequencies, the thresholds were comparable among seals, showing a range in sensitivity of 13 dB for all animals. Exceptions occurred at 1, 2 and 20 kHz, where differences in hearing thresholds varied from 25 to 35 dB. Hearing sensitivity declined rapidly below 3 kHz at a rate of ∼40 dB/octave.

**Figure 6:**
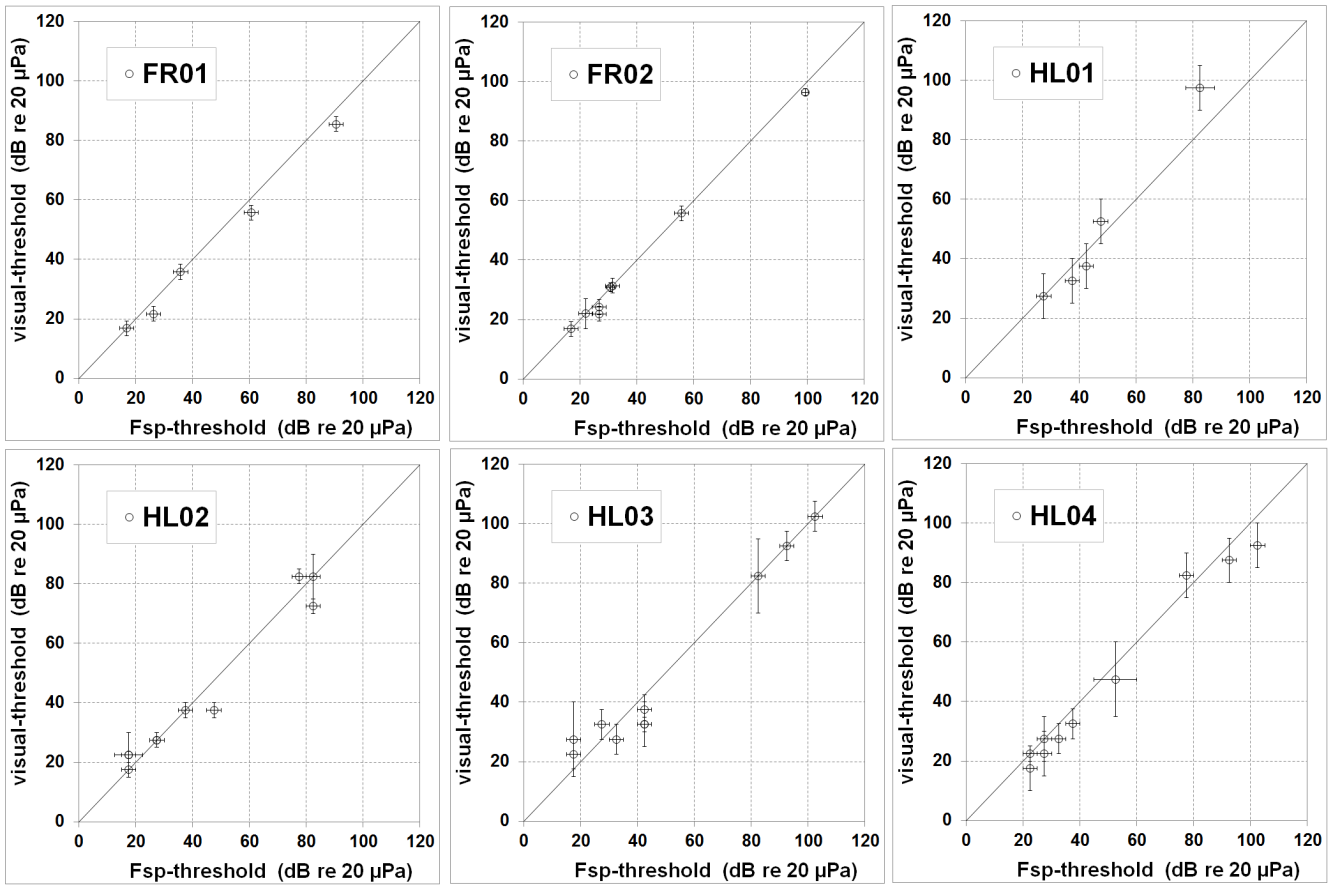
Comparison of the methods used for threshold detection. Comparison of ABR thresholds derived with an F_sp_-test and a visual analysis of the ABR waveform. The results show generally good agreement suggesting that the accuracy and variability in the visual and F_sp_ methods are comparable.

A comparison of the hearing thresholds estimated by visual inspection and by utilizing the F_sp_ is shown in Figure 6. Results of the F_sp_-test and the visual estimation of signal presence were in good agreement until threshold was reached; while the F_sp_-test shows that a waveform was detected at 37 dB SPL, the stereotypical wave V is not clearly visible within the waveform. Average differences between the thresholds were lower than 4 dB for most frequencies; exceptions occurred at 1.4 kHz, where the mean threshold determined visually was 5 dB lower than that determined with the F_sp_, and for 2.8 kHz, where it was 7 dB lower.

## Discussion

Information on hearing abilities of pinnipeds is limited or unavailable for most species. Within the phocid seals, aerial audiograms have been measured for the harp seal (*Pagophilus groenlandica,* [34]), harbour seal (*Phoca vitulina*, [35], [27], [36], elephant seal (*Mirounga angustirostris*, [23]) and grey seal (*Halichoerus grypus*, [26]). Except for the work by Ridgway and Joyce [26] and Wolski et al. [27], phocid seal threshold audiometry has been primarily conducted through behavioural methods. Behavioural methods are the standard for audiometry and ABR methods do not provide a direct assessment of the absolute threshold; rather, they provide an estimate of the threshold based on measureable neural signals produced by the auditory brainstem in response to audible sounds. Indeed, it is common for ABR threshold estimates to underestimate hearing sensitivity, particularly at the low and high ends of the audible range (e.g. see [37], [38]). In this study, we relied upon ABR methods in combination with earphone inserts to test the hearing capabilities of grey seals. The ABR methods were chosen to permit more rapid testing of hearing in a number of animals. The earphone inserts were used in an effort to overcome reports that phocid seals have the ability to manipulate a sphincter associated with the external auditory meatus, which inhibits reception of airborne sounds.

Ridgway and Joyce [26] reported the best sensitivity of grey seals to be ∼47 dB SPL at 4 kHz. Although there is difficulty in interpreting this finding because the method of calculating the stimulus is not sufficiently reported by Ridgway and Joyce [26], our results suggest that grey seal auditory thresholds estimated from tone pip ABRs are well below 30 dB SPL from 4 to 11.2 kHz and potentially below 40 dB SPL up to 20 kHz. In addition to some uncertainty in the received stimulus level reported by Ridgway and Joyce [26], a number of methodological considerations must also be considered when comparing the two studies. First, Ridgway and Joyce measured cortical evoked responses with relatively long duration stimuli (100 ms). In contrast, the evoked responses monitored in this study originated in the brain stem. As such, they are less affected by the attentive state of the animal and are more robust to physiological perturbation. The tone pip duration was also shorter in this study with a maximum duration of 5 ms. The shorter duration tone pip has a greater bandwidth (see Figure 3) and produces a robust ABR with a potentially lower threshold of detection. Likewise, it cannot be ruled out that the seals used by Ridgway and Joyce [26] affected their acoustic exposure by either their movement or the manipulation of the sphincter surrounding the external ear. Indeed, Ridgway and Joyce [26] report that seal movement relative to their sound projector was problematic. Although the use of earphone inserts has its own challenges, the combined use of earphone inserts and chemical immobilization of the seals should have reduced physiological noise and helped to stabilize the acoustic field during testing.

Within the range of frequencies tested, our results have some similarities to in-air hearing tests of other phocid seals above 3 kHz (Figure 5). Below 3 kHz there is a steep reduction in hearing sensitivity, with a higher offset compared to results of psychoacoustic studies. The reduction in sensitivity at frequencies below 3 kHz relative to prior behavioural studies is not unexpected given fundamental differences in the two methodologies applied. Similar differences were noted by Wolski et al. [27] in their comparison of ABR and behavioural hearing thresholds below 8 kHz in the harbour seal. However, a direct comparison cannot be made to that study as Wolski et al. [27] measured thresholds as a function of the stimulus energy, not the sound pressure level (as was done here). To determine how representative the ABR data collected here are to behavioural hearing thresholds in grey seals, a comparison similar to that performed by Wolski et al. [27] should be performed with the grey seal.

Although the results of Wolski et al. [27] are the most relevant data to compare to the current study, it should be noted that lower hearing thresholds have been obtained for the harbour seal when tested in an anechoic chamber [39]. Results obtained from studies conducted in an anechoic chamber, which mitigates potential masking due to ambient noise, have shown that thresholds can be overestimated if audiometry is performed in an environment where the potential for masking is not controlled. Most of the studies to which the results of this study were compared (Figure 5) were done in outdoor environments and were probably affected by some degree of masking. Nevertheless, although testing in outdoor environments can impede the ability to determine absolute thresholds, the masking conditions are more likely to represent conditions that the seals face in their natural environment.

The use of earphone inserts has advantages over the use of direct field stimulation. In untrained animals in which ABRs are measured, the use of earphone inserts can mitigate the closing of the sphincter of the external acoustic meatus. Earphone inserts may also provide better attenuation of background noise than headphones. For example, the seal that was tested in the presence of the highest background noise levels (Figure 1D) demonstrated a hearing sensitivity comparable to other grey seals tested in environments with less background noise. The comparable results may be due to the ear inserts attenuating background noise levels; however, a precise evaluation of how well the earphone inserts attenuate background noise remains to be completed.

The earphone inserts are not without problems. The loss of signal fidelity in projected tone pips at higher frequencies is a concern. The loss of signal fidelity at the higher frequency stimuli is due to the interaction of impulsive nature of the tone pip, the dimensions of the ear insert, and the transfer function of the in-ear headphones. Further calibrations with inserts of differing lengths showed that varying the insert length also varied the fidelity of the signal. Conversely, projection of tonal signals through the inserts, including sinusoidal amplitude modulated tones, had high signal fidelity. Thus, the inserts may be better suited for obtaining auditory steady state responses (ASSR) with amplitude modulated tones; however, this comes at a cost as the use of more tonal signals will reduce the amplitude of the evoked response. Furthermore, although calibration of the earphone insert was made with an artificial ear calibrator, it is acknowledged that this may not adequately represent the ear canal of the seal. Future efforts should develop methods of calibrating the earphone inserts in place, as has been done in other pinniped ABR studies with headphones [40].
